# Digital Forensics of Scanned QR Code Images for Printer Source Identification Using Bottleneck Residual Block

**DOI:** 10.3390/s20216305

**Published:** 2020-11-05

**Authors:** Zhongyuan Guo, Hong Zheng, Changhui You, Xiaohang Xu, Xiongbin Wu, Zhaohui Zheng, Jianping Ju

**Affiliations:** 1 School of Electronic Information, Wuhan University, Wuhan 430072, China; guozhongyuan@whu.edu.cn (Z.G.); youchanghui@whu.edu.cn (C.Y.); xuxiaohang@whu.edu.cn (X.X.); xbwu@whu.edu.cn (X.W.); gjdxjjp@whu.edu.cn (J.J.); 2 School of Mathematics and Physics, Wuhan Institute of Technology, Wuhan 430079, China; zhengzhaohui@whu.edu.cn

**Keywords:** digital image forensics, QR code, printer source identification, bottleneck residual block

## Abstract

With the rapid development of information technology and the widespread use of the Internet, QR codes are widely used in all walks of life and have a profound impact on people’s work and life. However, the QR code itself is likely to be printed and forged, which will cause serious economic losses and criminal offenses. Therefore, it is of great significance to identify the printer source of QR code. A method of printer source identification for scanned QR Code image blocks based on convolutional neural network (PSINet) is proposed, which innovatively introduces a bottleneck residual block (BRB). We give a detailed theoretical discussion and experimental analysis of PSINet in terms of network input, the first convolution layer design based on residual structure, and the overall architecture of the proposed convolution neural network (CNN). Experimental results show that the proposed PSINet in this paper can obtain extremely excellent printer source identification performance, the accuracy of printer source identification of QR code on eight printers can reach 99.82%, which is not only better than LeNet and AlexNet widely used in the field of digital image forensics, but also exceeds state-of-the-art deep learning methods in the field of printer source identification.

## 1. Introduction

With the popularity of smart phones and 4G networks, QR codes are widely used in logistics and transportation, financial institutions, advertising and marketing, security, and anti-counterfeiting [1,2,3,4,5]. It records data symbol information according to certain rules and distributes black and white images on a plane, and it has the advantages of low printing cost, easy production, and durability.

At present, printers are office equipment commonly used by institutions, enterprises and individuals, and digital printing is also widely used in people’s daily lives. As an important part of digital content, QR codes are usually printed on newspapers, magazines, advertisements, books, packaging, personal business cards, and other carriers. Users can scan the QR codes with a mobile phone camera to achieve the purpose of information acquisition, website redirection, advertisement push, anti-counterfeiting traceability, mobile payment, information technology, etc. [6,7,8,9] that uses the QR codes as the entrance has attracted more and more mobile phone users and businesses, and has become the current development trend. However, the QR code has the risk of being forged during the printing process, which is likely to cause economic disputes and criminal cases, resulting in huge economic losses and serious crimes. Therefore, it is necessary to identify the source of the printer. In the printing process of digital content, drifting motor and precision gear have created pattern information from the printer as the inherent information of device, so that each printer has a characteristics signature based on the corresponding fluctuation of the developed toner on the printed page, and each printer has its own unique texture features [10]. According to the above principles, the source forensic of printed documents can be carried out.

The currently widely used printed source identification techniques are pattern based shallow learning [11,12,13]. Tsai [14] et al. used Grey-Level Co-occurrence Matrix (GLCM) and discrete wavelet variation methods to extract the characteristics of scanned Chinese characters to find the printer source; Choi [15] et al. used noisy texture analysis and support vector machine (SVM) to detect the printer source of unknown images; Zhou [16] et al. proposed a text-independent printed identification method based on texture synthesis, Fast Fourier Transform (FFT) and GLCM were used for feature extraction, and SVM was used for classification. Similar methods have been developed by Mikkilineni [17], Gebhardt J [18], and Fang T [19]. However, the above methods require the participation of experts in the process of feature extraction, feature selection and classifier design. In addition, the entire process has to be repeated several times to obtain generalized results, which will undoubtedly consume time.

In recent years, deep learning has been widely used, and CNN has also made breakthroughs in image classification, image recognition and other issues [20,21,22,23,24,25,26]. As a new field of machine learning, deep learning uses the structure of a deep neural network with multiple convolution layers, which automatically learns the features of large-scale input data, and forms the bottom features into more abstract high-level features to represent the attribute categories or features of training data, and then represents the attribute categories or features of training data, so as to achieve higher recognition accuracy. Different from the traditional pattern based shallow learning, the structure of deep learning model contains more layers, and the number of hidden layers is usually above 5. Furthermore, deep learning can extract features layer by layer and transform data samples in the original feature space into a new feature space to represent the initial data, making classification or prediction problems easier to implement. Comparing with manually designed feature extraction methods, the data features obtained by deep learning models can better represent the rich internal information of big data.

In the studies of printer source identification based on deep learning, scientists and researchers often directly introduce CNNs which are commonly used in the field of computer vision to classify printers. However, there are essential differences between the printer source identification and classification in computer vision. In the field of computer vision, the morphological differences between categories are larger, which can be distinguished by human eyes; but in the field of printer source identification, the morphological differences between categories are extremely small, and the differences between categories exist in a weak form [27]. At present, the commonly used CNNs in the field of printer source identification of digital documents are generally designed based on LeNet [28] or AlexNet [29], because compared with VGGNet [30], GoogLeNet [31], ResNet [32], and other CNNs with more layers, they have lower structural complexity, have better tentative conditions for forensic problems with fewer data sets, and are more suitable for small-size image input.

In addition to the above general classical CNNs, researchers have also proposed a series of printer source identification methods of digital documents based on deep learning. Anselmo Ferreira [10] designed a deep learning method for laser printer attributes based on the analysis of small patches representing text characteristics. The proposed method uses multiple CNNs to learn multiple representations of the same character, and then the features are fused to improve the identification accuracy. However, this method will undoubtedly consume a lot of time and energy in the process of data collection and processing. Do-Guk Kim [33] proposed a cascaded learning method based on generative adversarial networks (GANs) and convolutional neural networks (CNNs) to identify the source color laser printer of photographed color documents. This method uses Simulated + Unsupervised (S + U) learning based on a GAN-alike framework for the decomposition of halftone color channels and proposed a CNN for source color laser printer identification, but it cannot be used for printer source identification of scanned images, because there are differences in the intensity of the illumination and clarity between the photographing environment and the scanning environment. Min-Jen Tsai [34] designed a CNN with seven layers to identify the printer source of text and images collected under microscopes and scanners, so as to achieve the same effect as pattern-based shallow learning, and effectively avoided the shortcomings of pattern based shallow learning method of manually designing feature extractor. However, there was no detailed analysis and discussion on the design of CNN structure in the field of printer source identification, such as the data input size of the network, the selection of the kernel size in the convolutional layer, etc.

The above forensic methods are mainly designed for grayscale text documents or color-captured image, but there are few studies on the source printer identification of widely used black and white QR codes. In addition, previous work rarely introduces breakthrough structures in deep learning into the field of printer source forensics, and achieves excellent identification results while ensuring low structural complexity of CNNs. Therefore, it is a work of great research value and practical significance to apply the widely and far-reaching structure of deep learning to the field of printed source identification of QR codes simply and effectively, at the same time, it has also become an urgent requirement for the legality of identification evidence in criminal and civil case in the judicial system.

In this study, a printer source identification network (PSINet) based on BRB, which is customized for the black and white image of the QR code, is proposed, and exploratory experiments have been done in terms of data input size, convolution kernel size, and CNN architecture; its purpose is to obtain the highest and most stable identification effect. Note that we do not limit the specific content of the QR code, and we assume that one of all the printers we use is a certified official printer and that the other printers may come from the companies different from the official printer, or they may come from the same company where the certified official printer is located, but the specific models are different.

The structure of this paper is as follows. Section 2 introduces the materials and method. Section 3 is the experimental results and analysis. Section 4 summarizes the paper.

## 2. Materials and Methods

### 2.1. The Printer Source Identification of QR Codes Using CNNs

**Figure 1:**
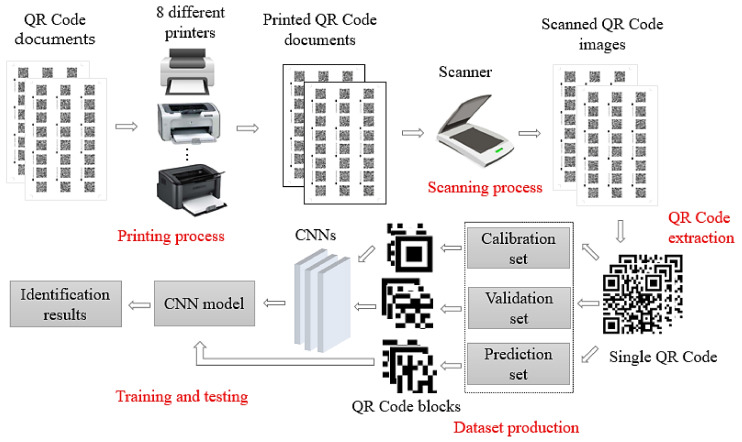
The overall flow chart of printer source identification process of QR codes using convolution neural networks (CNNs).

The diagram shown in Figure 1 illustrates the printer source identification process of QR codes using CNNs, which can be divided into five stages such as printing process, scanning process, QR code extraction, dataset production, and training and testing of CNNs.

(1) Printing process. Eight printers were used to print the same batch of QR code documents.
(2) Scanning process. The printed QR code paper documents were scanned by the same scanner to generate digital images.
(3) QR code extraction. It can be seen from Figure 1 that there are many QR codes in the scanned digital images and a single QR code needs to be extracted. In this study, OpenCV, a cross-platform computer vision and machine learning software library based on Berkeley Software Distribution (BSD) license, is used; the main extraction steps include graying, binarization, morphological opening operation, image reverse operation, finding contours, and setting the threshold range to extraction QR code contour area. These operations are well known in the field of image processing and are described in detail in Section 2.2.
(4) Dataset production. Adjust the size of all the extracted QR codes to 256 × 256, and then make the calibration dataset, validation set, and prediction set in the ration of 3:1:1. Then, block all the QR code images in the data set to a size of M × N.
(5) Training and testing of CNNs. Design PSINet, then the QR code blocks are input for training and testing.

### 2.2. QR Code Extraction

(1) Grayscale.

The scanned QR code images have three channels: *R*, *G*, and *B*, so it needs to be grayed. The human eye has the highest sensitivity to green and lowest sensitivity to blue. Therefore, the weighted average method is used to weight the RGB components of QR code image with a certain weight to obtain a reasonable gray image. The Open Source Computer Vision Library (OpenCV) is used, and the grayscale function is as follows,

(1) Gray(i,j)=0.299×R(i,j)+0.587×G(i,j)+0.114×B(i,j)

where *R*, *G*, and *B* represent the brightness of red, green, and blue channels, respectively.

(2) Otsu’s segmentation.

Otsu method can adaptively segment the QR code and background according to the gray characteristics of the image to maximize their inter-class variance. The threshold in the experiment is set to 127 to obtain a good segmentation effect.

(3) Morphological opening operation.

The process of first corrosion and then expansion is adopted, which can remove isolated connected domains and fill the inside of the QR Code. The OpenCV library is used and the shape of the kernel of the structure element is set to rectangle, and the size of the kernel is 30 × 30.

(4) Image reversal.

The formula of image reversal is as follows,

(2) g(x,y)=255−f(x,y)

where *f(x, y)* represents the gray value of *(x, y)* and *g(x, y)* represents the gray value of *(x, y)*; after image reversal, the QR code is changed into white and the background area is changed into black.

(5) QR code extraction.

The OpenCV library is used to find the contour of the QR codes, and the QR code is extracted by setting the threshold of the area and height of the contour. The formula is as follows,

(3) $$Rect_{area}>20,000,\;100<Rect_{height}<500$$

where $Rect_{area}$ represents the area of the contour of QR code and $Rect_{height}$ represents the height of the contour of QR code.

### 2.3. PSINet: A Printer Source Identification Network for QR Codes Based on Bottleneck Residual Block

CNN is mainly composed of convolution layers, pooling layers, ReLU layers, and fully connected layers. When designing a CNN, multiple convolution layers are often used to extract deep features of images, and batch normalization layer is generally fused with the convolutional layer, and its main function is to alleviate the vanishing gradient and gradient explosion, and to accelerate the training speed. Pooling layer reduces the dimensionality of the feature map while maintaining important information, it is sandwiched between continuous convolutional layers to compress the amount of data and parameters, thereby preventing overfitting. The function of ReLU layer is to perform nonlinear mapping of the results of convolutional layer to increase the nonlinear expression ability of CNN. The fully-connected layer serves as the classifier in the entire CNN and represents the learned distribution features into the sample label space.

**Figure 2:**
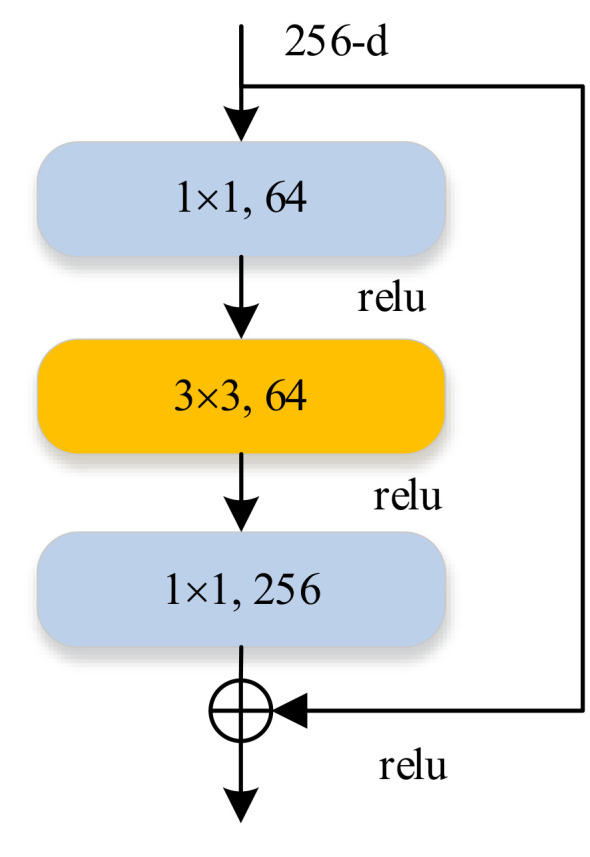
Bottleneck residual block (BRB).

In 2015, He Kaiming, a researcher at Microsoft Research Institute, proposed a residual network with 152 layers and won the championship in the image recognition project of the ImageNet Large Scale Visual Recognition Challenge (ILSVRC). The residual network proposed the bottleneck residual block (BRB) structure, as shown in Figure 2. BRB is composed of 1 × 1 convolution layer, ReLU layer, 3 × 3 convolution layer, ReLU layer, 1 × 1 convolution layer, ReLU layer. The number of feature maps of the three convolutional layers in BRB is 64, 64, 256, respectively. By introducing identify mapping into CNN, the problem of gradient diffusion in deep CNN can be effectively solved, and the parameters are reduced while the depth of CNN is increased, which further improves the performance of CNN.

**Figure 3:**
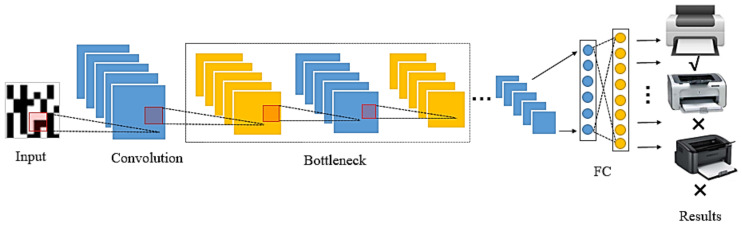
The identification process of PSINet.

In this study, we were inspired by a lot of research work on the application of residual networks in the field of computer vision. At the same time, considering that BRB has a strong feature representation capability, we first introduced the BRB into the field of printer source identification of digital content, and designed a CNN named PSINet to identify the printer source of QR codes. Figure 3 is the overall framework of PSINet, which mainly follows the structure of convolution layer, pooling layer, BRBs, pooling layer, and fully connected layer. Each convolutional layer is followed by a batch normalization (BN) layer and a ReLU layer. The number of BRBs and the final structure of PSINet will be determined through the experiment. It is worth mentioning that the global average pooling layer is used before the fully connected layer, which can effectively prevent overfitting. The size of the convolutional kernel of the global average pooling layer is determined according to the size of feature map output by the previous convolution layer, and stride is 1; the sizes of the other pooling layers are 5 × 5, and the strides are 2; as the number of printers used is 8, the output categories of the fully connected layer is set to 8; finally, the softmax layer maps the multiple scalars output by the fully connected layer into probability distributions. The formula is as follows,

(4) $$f(z_{i})=\frac{e^{z_{i}}}{\sum_{i=1}^{n}e^{z_{i}}}$$

where $Z_{i}$ represents input, n represents categories, and $f(z_{i})$ represents output.

The hyperparameter settings of PSINet are as follows; training epoch is set to 30 and the number of iterations per epoch is 576; therefore, a total of 7280 iterations are performed, which can ensure that training curve fully converges; the solver type is set to stochastic gradient descent (SGD); the base learning rate is set to 0.01, the policy is set to exponential decay, and gamma is set to 0.99; momentum is set to 0.9 and weight decay is set to 0.0001.

## 3. Experimental Results and Analysis

### 3.1. Development Environment and Tools

We used a personal computer (Intel^®^ Core^TM^ i7-7700HQ CPU @2.80 GHz (Intel Corporation, Santa Clara, CA, USA), GeForce GTX 1070 (NVIDIA Ltd., Santa Clara, CA, USA) and 16 GB RAM with Windows10 operating system) to develop the proposed PSINet; the software development environment of QR code extraction and segmentation program is Visual Studio 2015 (Microsoft Ltd., Chicago, IL, USA) and OpenCV-3.4.0 (Intel Ltd., Santa Clara, CA, USA); the deep learning framework used is Caffe (Berkeley Artificial Intelligence Research, San Francisco, CA, USA) [35] and Nvidia Digits-6.1.1 (NVIDIA Ltd., Santa Clara, CA, USA) [36].

### 3.2. Dataset Production

**Figure 4:**
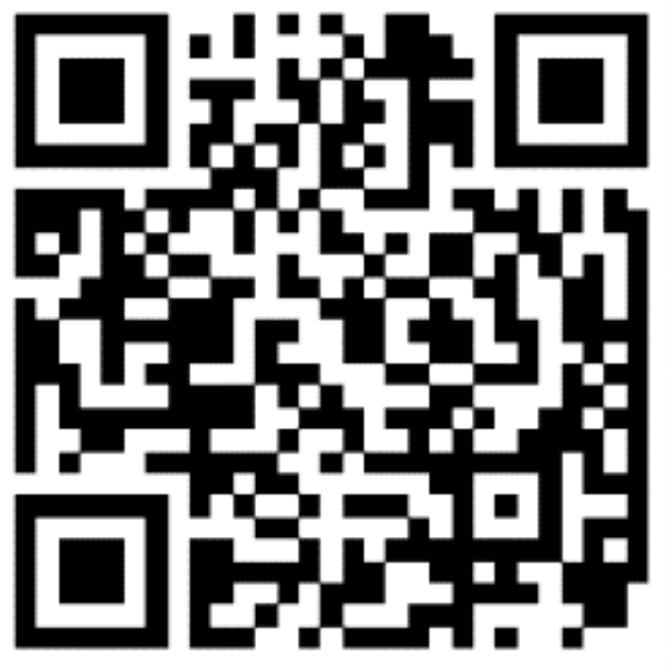
The extracted QR code image.

**Figure 5:**
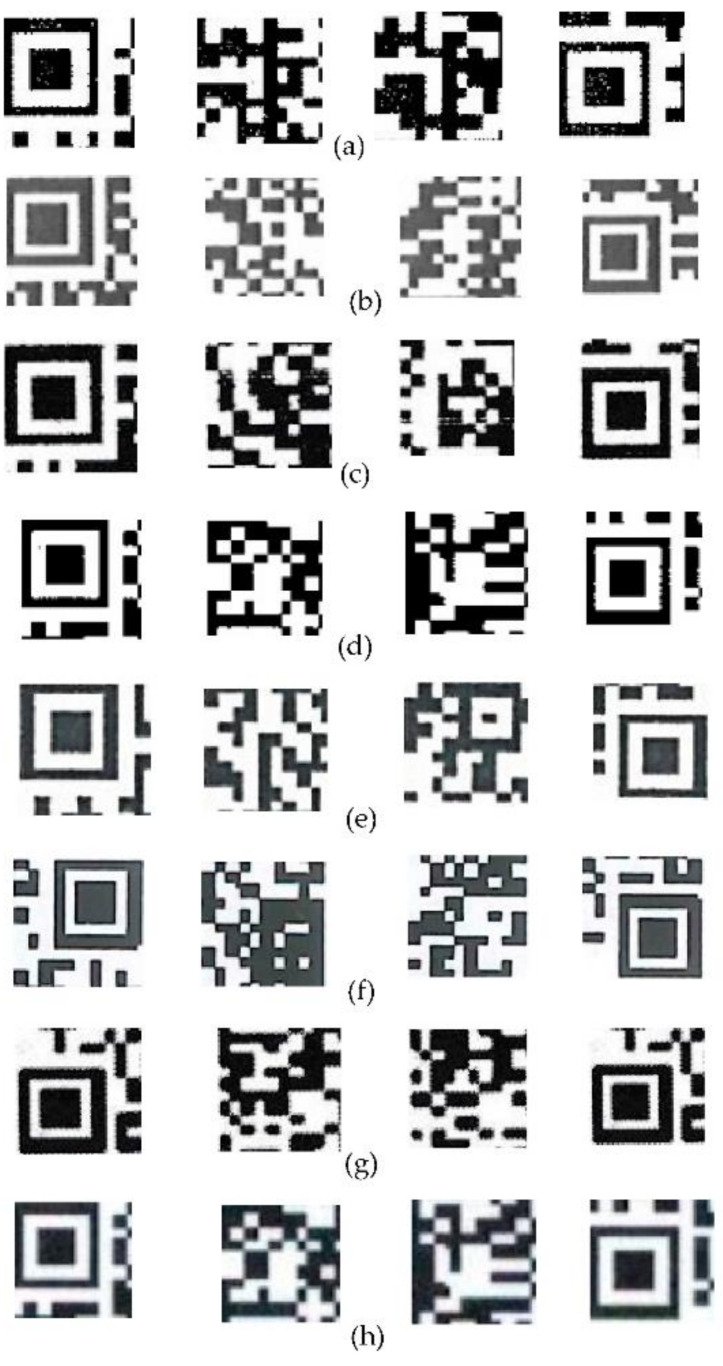
Image samples for QR codes printed by eight printers. (**a**) Canon iR-ADV C50455051 UFR2, (**b**) Canon iR-ADV C7260270 UFR2 (Canon Inc., Tokyo, Japan), (**c**) Epson L310 Series(Seiko Epson Ltd., Suwa, Japan), (**d**) Fuji XEROX DocuCentre S2110, (**e**) RICOH Aficio MP7502(Ricoh Ltd., Tokyo, Japan), (**f**) RICOH Pro8100s, (**g**) Samsung K2200 series(Samsung Electronics Ltd., Seoul, Korea), and (**h**) TOSHIBA e-STUDIO2051C-11606695(Toshiba, Tokyo, Japan).

The extracted single QR code sample is shown in Figure 4, then the QR code data set are made. In this study, 8 printers are used; the specific brands and models are shown in Table 1. The number of QR codes is 240, after printing by 8 printers, the total number becomes 1920, and they are scanned into digital images by the same scanner with a resolution of 300 dpi. Then, the sizes of all QR codes are unified to 256 × 256, and the calibration set, validation set, and prediction set are made according to the ratio of 3:1:1, and each QR code image is divided into blocks with size of M × N. Figure 5 shows the block images of some QR codes printed by eight printers.

### 3.3. Determination of the Specific Structure of PSINet

First of all, when CNN is applied to the printer source identification of digit content, the image input size will have an impact on the performance of CNN to extract the inherent micro-features in the printing process. In this study, the printed and scanned QR code images are divided into blocks with sizes of 48 × 48, 56 × 56, and 64 × 64, respectively, and the block size that is most suitable for the QR code printer source identification is determined through experimental comparison. Then, the size of the convolution kernel also has an important influence on the accuracy of CNN. It can be seen from the application of residual network in the field of computer vision that when the BRB architecture is stable, setting the convolution kernel size of the first convolution layer to 7 × 7 has a better effect. In the field of printer source identification, the input image size is usually small, and the differences between categories are relatively subtle. Correspondingly, the impact of the size of the convolution kernel in the first convolutional layer on image feature extraction ability also requires experimentation. In this study, convolutional kernels with size of 3 × 3, 5 × 5, 7 × 7 were selected respectively. Finally, the number of CNN layers also has a very important impact on the identification accuracy. PSINet is mainly designed based on BRB, in this paper, we set the number of BRBs to 1, 2, and 3, respectively, at the same time, and the layers of PSINet are 7, 11, and 15, respectively. The identification accuracy of PSINet under different image input sizes, different convolution kernel sizes of first convolution layer, and different convolution layers is shown in Table 2.

It is well known that a single dataset is not representative, so in order to determine the size of QR code image block, the convolutional kernel size of the first convolution layer and the number of layers of PSINet, we use two indicators of average accuracy and standard deviation to determine the final parameter size. The specific process is as follows.

When the image size is 48 × 48, 56 × 56, 64 × 64, the average accuracy is (99.03 ± 1.26)%, (99.44 ± 0.66)%, and (99.56 ± 0.47)%, respectively. It can be seen that when the image block is 64 × 64, the average accuracy is the highest, and the standard deviation is the smallest, indicating that the proposed PSINet has the best identification performance and stability. Therefore, the QR code image block with size of 64 × 64 is selected as the input.

Then, the size of the convolution kernel of the first convolution layer is determined through experiments. When the size of the convolutional kernel is 3 × 3, 5 × 5, 7 × 7, respectively, the average accuracy is (99.37 ± 0.86)%, (99.33 ± 0.61)%, and (99.33 ± 0.99)%, respectively. It can be seen that when the size of the convolution kernel is 3 × 3, the average accuracy is the highest, which is 0.04% higher than the 5 × 5 convolution kernel and 7 × 7 convolution kernel, but the advantage is very weak; on the contrary, the difference in standard deviation is obvious: the standard deviation of the 5 × 5 convolution kernel is the smallest, with a value of 0.61%, which is 0.25% and 0.38% lower than 3 × 3 convolution kernel and 7 × 7 convolution kernel, respectively. The above analysis shows that while the 5 × 5 convolution kernel obtains similar accuracy, its stability can be significantly better that other convolution kernels, so it is selected.

Finally, the number of layers of PSINet is determined. When the number of layers of PSINet is 7, 11, and 15, the average accuracy rates are (99.81 ± 0.14)%, (99.69 ± 0.12)%, and (98.52 ± 0.95)%, respectively; it can be seen that when the number of layers of PSINet is 7 layers, the average accuracy is the highest, which is 0.12% and 0.29% higher than that of 11 layers and 15 layers, respectively; in terms of standard deviation, the standard deviation of PSINet with 11 layers is the smallest, which is 0.12%, but PSINet with 7 layers obtained a very close value of 0.14%.The PSINet with 15 layers performs the worst in terms of average accuracy and standard deviation. In summary, PSINet with 7 layers is selected.

It can also be seen from the experimental results that if we choose Canon iR-ADV C50455051 UFR 2 as the official printer, regardless of whether the suspected printer is from Canon’s other models, such as Canon iR-ADV C7260270 UFR 2, or from other companies’ printers, such as Epson L310 Series and RICOH’s Aficio MP7502, the PSINet we proposed can effectively identify.

### 3.4. Comparison of PSINet with Other Deep Learning Methods

#### 3.4.1. Accuracy Comparison of Different CNNs

In this section, PSINet is compared with other CNN methods in identification accuracy to verify its effectiveness. It is well known that in the field of printer source identification, the size of input image is relatively small, and it can be seen from Section 3.3 that the shallow convolutional neural network is more suitable for printer source identification. Based on the above considerations, LeNet and AlexNet, which are currently widely used in the field of digital image forensics, are selected for comparison. In addition, there is CNN method specifically proposed for the field of printer source identification, such as Min-Jen Tsai’s [28], which is also used for comparison to verify the effectiveness and superiority of PSINet. It is worth mentioning that Anselmo Ferreira’s [26] and Do-Guk Kim’s [27] are not considered because their data input methods and data collection scenarios are quite different from that of PSINet. The size of the QR code image blocks used in the experiment are 64 × 64, after the blocks are divided according to the above size, there are 18,432 images in the calibration set, 6144 images in the validation set and 6144 images in the prediction set. They are evenly distributed in 8 types of printers, that is, each printer prints the same amount.

**Figure 6:**
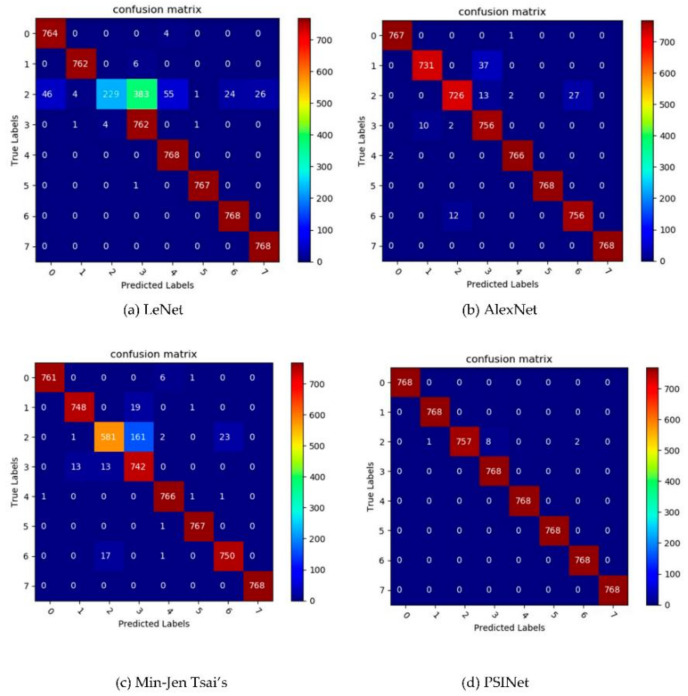
Graphical confusion matrices of several CNN methods.

First, a confusion matrix is used to measure the accuracy of classification model, the confusion matrix of LeNet, AlexNet, Min-Jen Tsai, and PSINet is shown in Figure 6, where the columns represent predicted labels and the rows represent true labels, the values on the diagonal indicates the number of correct prediction, and non-diagonal elements are the part of prediction errors. The higher the value on the diagonal of the confusion matrix, the more accurate the prediction result. It can be seen that the value on the diagonal of the confusion matrix of PSINet is the largest, followed by AlexNet, then Min-Jen Tsai’s, and finally LeNet. Among them, PSINet has a high number of correct identification on all eight types of printers, while the number of correct identification of AlexNet on class 1 and class 2 printers in slightly lower than that of PSINet, the performance of Min-Jen Tsai’s on class 2 printer is significantly lower than that of PSINet and AlexNet, only 581 were correctly identified, LeNet’s test on class 2 printer is undoubtedly the worst, only 229 were correctly judged.

**Figure 7:**
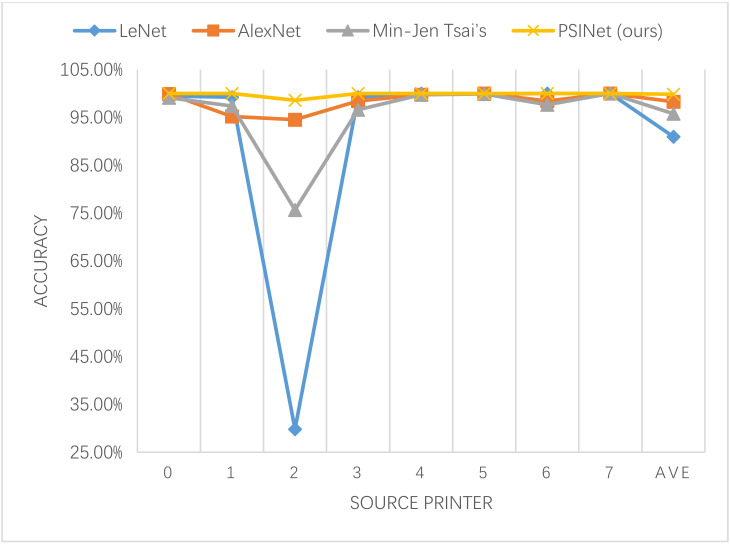
Comparison of the accuracy of the four CNN methods.

In addition to using the confusion matrix to represent the number of correct and wrong predictions for each category, the accuracy index is also used. The accuracy distributions of four CNNs are show in Figure 7 and Table 3, which, respectively, reflect the identification accuracy of each type of printer and average accuracy. In terms of the identification accuracy of each type of printer, PSINet performed better than LeNet, AlexNet, and Min-Jen Tsai’s in class 0, 1, 2, 3, 4, and 5 printers. The performance on the 6th printer is the same as LeNet, reaching 100.00%, 1.56% higher that AlexNet and 2.34% higher than AlexNet. The identification accuracy of four CNN methods on the 7th printer all reach 100.00%. In terms of average recognition accuracy, the identification accuracy of LeNet is 90.95%, that of AlexNet is 98.27%, and that of Min-Jen Tsai’s is 95.75%, PSINet achieves 99.82%, which is better than LeNet, AlexNet, and Min-Jen Tsai’s by 8.87%, 1.55%, and 4.07%, respectively. The effectiveness and superiority of PSINet are verified.

#### 3.4.2. Comparison of Inference Time of Four CNN Models

This section mainly compares the inference time used by the four CNNs on the prediction set. Generally speaking, in the case of the same amount of data and the same accuracy, the less the inference time, the better the performance of the CNN model. The prediction set has 6144 QR code blocks with 64 × 64 sizes, the result is shown in Table 4.

It can be seen from Table 4 that the inference time of Min-Jen Tsai’s method is 11 s, which is the least among the four CNN methods; then, there is LeNet and PSINet, their inference time is 13 s; AlexNet has the longest inference time, which is 24 s. Combined with the identification accuracy, it can be seen that although the inference time of PSINet is 2 s longer than that of Min-Jen Tsai’s, the identification accuracy is 4.07% higher; PSINet and LeNet have the same inference time, but the identification accuracy of PSINet is 8.87% higher than that of LeNet; the inference of PSINet is 11 s less than that of AlexNet, and the identification accuracy of PSINet is 1.55% higher than that of AlexNet. Therefore, the performance of PSINet is quite excellent.

#### 3.4.3. Comparison of Computational Cost of Four CNN Models

This section is a comparison between the computational costs of four CNN models, and their model sizes are shown in Table 5. The large the CNN model, the greater the number of parameters, and the higher the computational cost. It can be seen that the model sizes of LeNet, AlexNet, Min-Jen Tsai, and PSINet are 16.2 MB, 77 MB, 0.43 MB, and 5.18 MB, respectively. Comprehensive analysis shows that the model size of Min-Jen Tsai is the smallest, about one-twelfth of that of PSINet, and its average accuracy is 4.07% lower than that of PSINet; the model size of LeNet is 3.12 times that of PSINet, but the average accuracy is 8.87% lower than that of PSINet; the model size of AlexNet is 14.85 times that of PSINet, and the accuracy is 1.62% lower than that of PSINet. Overall, PSINet’s performance in terms of computational cost is still relatively good, but further optimization is worth encouraging.

## 4. Conclusions

Aiming at the problem that QR codes are easily copied and forged in the printing process, a printer source identification method of QR code image blocks based on convolution neural network (PSINet) is proposed in this paper. PSINet innovatively introduces the bottleneck residual block (BRB) into the field of printer source identification, and the data input, the size of the convolution kernel, and the CNN architecture are compared and analyzed in detail. Eight printers are used for experiments, and the results showed that the PSINet proposed in this paper has better performance than other CNNs.

With current solutions to the printer source identification of scanned QR code images achieving high classification results, we believe that it is time to meet greater challenges. For instance, in the case of digital camera shooting, the pattern noise of the camera itself, as well as the blur and reflection that often appear during the shooting process, will make the effect recognition difficult. The related printer source identification problem remains to be studied in the future. In addition, we also plan to experiment on other forms of documents such as color images and grayscale text. Finally, as the service life of printer increases, its internal mechanical components, such as precision gears and drifting motors, will also wear out, causing changes in the characteristic signature of the printer that may affect the identification results. We plan to investigate the above issue in the future.

## Figures and Tables

**Table 1:** Printer brand and models used in this study.

No.	Brand	Model
0	Canon	iR-ADV C50455051 UFR 2
1	Canon	iR-ADV C7260270 UFR 2
2	Epson	L310 Series
3	Fuji	XEROX DocuCentre S2110
4	RICOH	Aficio MP7502
5	RICOH	Pro 8100s
6	Samsung	K2200 series
7	TOSHIBA	e-STUDIO2051C-11606695

**Table 2:** The printer identification accuracy of PSINet under different image input sizes, different convolution kernel sizes, and different convolution layers.

Image Size	7 Layers			11 Layers			15 Layers		
	3 × 3	5 × 5	7 × 7	3 × 3	5 × 5	7 × 7	3 × 3	5 × 5	7 × 7
**48** **×** **48**	99.90%	99.49%	99.77%	99.84%	99.77%	99.59%	97.75%	98.41%	96.76%
**56** **×** **56**	99.92%	99.89%	99.93%	99.85%	99.71%	99.66%	98.00%	98.93%	99.06%
**64** **×** **64**	99.89%	99.82%	99.71%	99.46%	99.63%	99.72%	99.69%	98.36%	99.73%

**Table 3:** The accuracy of the four CNN methods.

Methods	Identification Accuracy (%)								
	0	1	2	3	4	5	6	7	Ave
**LeNet**	99.48%	99.22%	29.82%	99.22%	100.00%	99.87%	100.00%	100.00%	90.95%
**AlexNet**	99.87%	95.18%	94.53%	98.44%	99.74%	100.00%	98.44%	100.00%	98.27%
**Min-Jen Tsai’s**	99.09%	97.40%	75.65%	96.61%	99.74%	99.87%	97.66%	100.00%	95.75%
**PSINet (ours)**	100.00%	100.00%	98.57%	100.00%	100.00%	100.00%	100.00%	100.00%	99.82%

**Table 4:** Comparison of inference time of four CNNs.

CNNs	LeNet	AlexNet	Min-Jen Tsai’s	PSINet
**Inference Time(s)**	13	24	11	13

**Table 5:** Comparison of computational costs of four CNN models.

CNNs	LeNet	AlexNet	PSDI	PSINet
**Size (MB)**	16.2	77.0	0.43	5.18
